# Troponin in acute chest pain to risk stratify and guide effective use of computed tomography coronary angiography (TARGET-CTCA): a randomised controlled trial

**DOI:** 10.1186/s13063-023-07431-9

**Published:** 2023-06-13

**Authors:** Kuan Ken Lee, David Lowe, Rachel O’Brien, Ryan Wereski, Anda Bularga, Caelan Taggart, Matthew T. H. Lowry, Amy V. Ferry, Michelle C. Williams, Giles Roditi, John Byrne, Chris Tuck, Denise Cranley, Praveen Thokala, Steve Goodacre, Catriona Keerie, John Norrie, David E. Newby, Alasdair J. Gray, Nicholas L. Mills

**Affiliations:** 1 BHF Centre for Cardiovascular Science, University of Edinburgh, Edinburgh, EH16 4SA UK; 2 Department of Emergency Medicine, Queen Elizabeth University Hospital, NHS Greater Glasgow and Clyde, Glasgow, UK; 3 Department of Emergency Medicine, Emergency Medicine Research Group, Royal Infirmary of Edinburgh, Edinburgh, UK; 4 Institute of Cardiovascular and Medical Sciences, Glasgow University, Glasgow, UK; 5 Department of Cardiology, Queen Elizabeth University Hospital, NHS Greater Glasgow and Clyde, Glasgow, UK; 6 Edinburgh Clinical Trials Unit, Usher Institute, University of Edinburgh, Edinburgh, UK; 7 School of Health and Related Research (ScHARR), University of Sheffield, Sheffield, UK; 8 Usher Institute of Population Health Sciences and Informatics, University of Edinburgh, Edinburgh, UK

**Keywords:** High-sensitivity cardiac troponin, Computed tomography coronary angiography, Coronary heart disease, Acute coronary syndrome

## Abstract

**Background:**

The majority of patients with suspected acute coronary syndrome presenting to the emergency department will be discharged once myocardial infarction has been ruled out, although a proportion will have unrecognised coronary artery disease. In this setting, high-sensitivity cardiac troponin identifies those at increased risk of future cardiac events. In patients with intermediate cardiac troponin concentrations in whom myocardial infarction has been ruled out, this trial aims to investigate whether outpatient computed tomography coronary angiography (CTCA) reduces subsequent myocardial infarction or cardiac death.

**Methods:**

TARGET-CTCA is a multicentre prospective randomised open label with blinded endpoint parallel group event driven trial. After myocardial infarction and clear alternative diagnoses have been ruled out, participants with intermediate cardiac troponin concentrations (5 ng/L to 99th centile upper reference limit) will be randomised 1:1 to outpatient CTCA plus standard of care or standard of care alone. The primary endpoint is myocardial infarction or cardiac death. Secondary endpoints include clinical, patient-centred, process and cost-effectiveness. Recruitment of 2270 patients will give 90% power with a two-sided *P* value of 0.05 to detect a 40% relative risk reduction in the primary endpoint. Follow-up will continue until 97 primary outcome events have been accrued in the standard care arm with an estimated median follow-up of 36 months.

**Discussion:**

This randomised controlled trial will determine whether high-sensitivity cardiac troponin-guided CTCA can improve outcomes and reduce subsequent major adverse cardiac events in patients presenting to the emergency department who do not have myocardial infarction.

**Trial registration:**

ClinicalTrials.gov Identifier: NCT03952351. Registered on May 16, 2019.

**Supplementary Information:**

The online version contains supplementary material available at 10.1186/s13063-023-07431-9.

## Background

Patients with acute chest pain account for a tenth of all presentations to the emergency department and up to 40% of unplanned hospital admissions, with their assessment placing a major burden on health care resources [1–3]. Current assessment strategies focus on ruling in or ruling out acute myocardial infarction through clinical assessment, electrocardiography and serial cardiac troponin testing. Once myocardial infarction is ruled out, most patients are discharged from hospital without further investigation or treatment, even though a substantial proportion may have underlying coronary artery disease. Prior research suggests that use of high-sensitivity assays to quantify cardiac troponin concentrations within the normal reference range [4–8] can improve the risk stratification of patients with acute chest pain and may help clinicians identify those who would benefit most from further investigation and treatment.

Patients with cardiac troponin concentrations below the 99th centile upper reference limit on serial testing do not have myocardial infarction but may still have underlying coronary artery disease [9]. Unless these patients have ongoing pain or dynamic electrocardiogram changes, they are likely to be discharged from the emergency department [10] without a consistent approach to follow-up or further investigation. Although patients in whom myocardial infarction is excluded are at low risk of major adverse cardiovascular events in the short term [10], high-sensitivity cardiac troponin concentrations can refine risk prediction and identify those who may benefit from preventative therapies or coronary revascularisation [11].

We previously reported that patients with cardiac troponin I concentrations < 5 ng/L are at very low risk, with a negative predictive value of 99.6% for a composite endpoint of myocardial infarction or cardiac death at 30 days [5]. We have subsequently verified the effectiveness of this risk stratification threshold in an individual patient-level meta-analysis of 22,457 patients from nine countries, demonstrating that cardiac troponin I concentrations were < 5 ng/L in 49% of patients, of whom less than 5 per 1000 patients had a myocardial infarction or cardiac death at 30 days [8]. Incorporation of this risk stratification threshold into an early rule out pathway for patients with possible myocardial infarction increases the proportion of patients discharged safely from the emergency department by 21% [10]. However, patients without myocardial infarction and intermediate high-sensitivity cardiac troponin concentrations between 5 ng/L and the 99th centile upper reference limit are 10 times more likely to have a major cardiac event at 1 year compared to those with a concentration < 5 ng/L (5.3% versus 0.7%; adjusted odds ratio, 0.23 [95% confidence interval, 0.19 to 0.28]) [5, 8].

Similar approaches are now used in multiple accelerated diagnostic pathways [12, 13], and the use of separate risk stratification and diagnostic thresholds is now recommended by both the European Society of Cardiology (ESC) [14] and National Institute for Health and Care Excellence (NICE) guidelines [15]. Up to a third of patients have intermediate cardiac troponin concentrations, and the ESC guideline recommends clinicians consider further observation and investigation in these patients [11]. However, it is not clear what further investigation should be undertaken, and there is currently no evidence that any particular investigation can alter clinical outcomes.

We postulate that patients with acute chest pain and intermediate-risk high-sensitivity cardiac troponin concentrations will benefit from further investigation for coronary artery disease soon after discharge.

## Methods and design

### Study design

TARGET-CTCA is a multicentre prospective randomised open-label blinded endpoint (PROBE) parallel group event driven multicentre trial assessing superiority of computed tomography coronary angiography (CTCA) on the risk of myocardial infarction or cardiac death in patients with intermediate cardiac troponin concentrations discharged from the emergency department after exclusion of myocardial infarction compared to standard care. We recruited from emergency departments in 14 hospitals across 9 sites in the UK (Supplementary Table 1). The SPIRIT reporting guidelines was used (Supplementary file) [16].

### Study objectives

The primary objective of the study is to determine whether the use of high-sensitivity cardiac troponin testing to risk stratify and target CTCA improves outcomes in patients presenting to hospital with suspected acute coronary syndrome in whom myocardial infarction is ruled out. The secondary objectives are to determine whether this approach is safe and cost-effective and improves patients’ quality of life or symptoms.

### Primary endpoint

The primary endpoint is a composite of myocardial infarction or cardiac death. Myocardial infarction is defined according to the Fourth Universal Definition of Myocardial Infarction [17] whilst cardiac death is defined as death resulting from an acute myocardial infarction, sudden cardiac death, or death due to heart failure [18]. Both myocardial infarction and cause of death will be adjudicated by two independent clinicians blinded to trial intervention with any discrepancies resolved by a third clinician as described previously [19].

### Secondary endpoints

The secondary endpoints include clinical, patient-centred, process and cost-effectiveness. These are detailed in Table 1.

**Table 1:** Secondary endpoints

1. Clinical and patient-centred endpoints	• Myocardial infarction • Cardiac death • Cardiovascular death • Non-cardiovascular death • All-cause death • Unscheduled urgent coronary revascularisation • Hospital reattendance with suspected acute coronary syndrome • Proportion of patients with major bleeding (BARC 3–5) • Symptomatic status as assessed by the short form Seattle Angina Questionnaire (SAQ-7) up to 24 months of randomisation • Quality of life as assessed by the EQ-5D-5L up to 24 months of randomisation • Lifestyle changes including diet, smoking habit, and exercise at 24 months
2. Process endpoints	• Proportion of patients with allergy, anaphylaxis or acute kidney injury following study CTCA scan • Proportion of patients with clinically significant abnormal non-cardiac findings on study CTCA scan • Radiation dose from study CTCA scan • Proportion of patients undergoing non-study CTCA or invasive coronary angiography within 90 days of hospital attendance • Proportion of patients undergoing non-invasive stress test within 90 days of hospital attendance
3. Cost effectiveness endpoints	• Proportion of patients prescribed therapies for coronary artery disease • Proportion of patients undergoing non-invasive stress testing • Proportion of patients undergoing coronary revascularisation • Incremental cost per quality-adjusted life year (QALY) gained

*Abbreviations*: *BARC* Bleeding Academic Research Consortium definition of bleeding [20], *CTCA* computed tomography coronary angiogram

### Exploratory endpoints

Lifestyle changes including diet, smoking habit and exercise will be captured using a questionnaire 24 months following enrolment. This will be used to determine whether the intervention compared to standard care results in changes to lifestyle or risk factors. To explore the pathophysiological mechanisms that influence cardiovascular risk, blood samples will be obtained and stored from consented participants, and we will perform visual and quantitative assessment of atherosclerotic plaque identified on CTCA imaging.

### Patient population

This trial will recruit at least 2270 participants from the emergency department or an equivalent acute medical admissions unit in secondary or tertiary care hospitals across the UK who will be randomised 1:1 to CTCA plus standard of care or standard of care alone. The inclusion criteria will be as follows: (i) presented to hospital with symptoms of suspected acute coronary syndrome; (ii) age ≥ 18 years old; and (iii) a maximum high-sensitivity cardiac troponin I or T concentration between 5 ng/L and the 99th centile. Exclusion criteria will be as follows: (i) diagnosis of myocardial infarction during index presentation; (ii) clear alternative diagnosis or participant requires further inpatient clinical assessment; (iii) CTCA or invasive coronary angiogram within 1 year from hospital attendance; (iv) inability to undergo CT scanning, for example due to severe renal failure (estimated glomerular filtration rate < 30 mL/min/1.73 m^2^) or major allergy to iodinated contrast media; (v) current pregnancy or breast feeding; (vi) inability to give informed consent; (vii) further investigation for coronary artery disease would not be in the patient’s interest, for example due to limited life expectancy, quality of life or functional status; or (viii) previous randomisation into the trial.

### Participant selection and enrolment

Eligible patients will be identified by the usual clinical care team in the emergency department or an equivalent acute medical admissions unit or using the electronic medical records after discharge. To ensure that our trial is inclusive and representative of the broad patient population presenting to hospital with suspected acute coronary syndrome, potential participants who are not approached in hospital (e.g. due to out of hours attendance or study team being unavailable) will be identified using electronic medical records or through referral from the clinical team and contacted by telephone to invite them to take part in the study. For the duration of the study, the trial will be incorporated into the clinical pathway for the evaluation of patients with suspected acute coronary syndrome. As part of the standard of care clinical pathway, patients with suspected acute coronary syndrome who are discharged from the emergency department will receive an information leaflet. This contains information about chest pain, a summary of the study, contact details of the research team, and explains that potential participants might be contacted after discharge.

Patients who are consented in the hospital will be asked to provide written informed consent whilst patients who are contacted by telephone will be given the option to either attend a study visit to provide written informed consent in person or provide informed consent verbally over the telephone. All patients who are randomised to CTCA will have their consent reaffirmed at the scanning visit and will be asked to sign a consent form before any further research activities are carried out.

### Randomisation

Following consent, patients will be randomised (1:1), either to CTCA plus standard of care or standard of care alone. Randomisation will be conducted using a web-based computer-generated system to ensure allocation concealment, stratified by age (< 70 years versus ≥ 70 years), sex, site and previously diagnosed coronary artery disease. This is an open-label trial design, and the patient and clinical care team will not be blinded to the intervention. Once randomised, a letter will be sent to the participant’s general practitioner (GP) to inform them of their involvement in the study.

### Study assessments

Study participants who are randomised to standard of care will not have any further study visits. Those randomised to CTCA with standard of care will be invited for an outpatient CTCA as soon as possible after their initial hospital attendance (ideally within 4 weeks of randomisation).

### Initial hospital attendance, screening visit or telephone contact

During the initial emergency department or equivalent acute medical admissions unit attendance, study visit or telephone contact, the research team will review participants’ clinical history including cardiovascular risk factors, past medical history, standard clinical biochemical and haematological variables, high-sensitivity cardiac troponin concentrations and the 12-lead electrocardiogram. The Seattle Angina Questionnaire (SAQ-7) shortened Rose Angina and EQ-5D-5L questionnaires will also be administered at baseline.

### Scanning visit assessment

To optimise the quality of the CTCA scan images, participants with a heart rate greater than 60 beats per minute may be prescribed a beta-blocker, rate-limiting calcium channel antagonist or ivabradine to slow their heart rate, and sublingual glyceryl trinitrate will be administered according to local guidance. Prescribing, dispensing and administration of rate limiting medications will follow local protocol and procedures.

### Computed tomography coronary angiography

Participants randomised to CTCA will undergo electrocardiogram (ECG) gated CTCA using ≥ 64-multidetector scanner, according to the Society of Cardiovascular Computed Tomography guidelines. Prospective or retrospective ECG-gating will be used according to heart rate and local protocols [21]. Tube current, tube voltage and volume and rate of iodine-based contrast media will be optimised based on body-mass index. Cardiac and wide field-of-view reconstructions will be produced and transferred to post-processing software for analysis. Images will be reported according to the Society of Cardiovascular Computed Tomography guidelines [22]. Per segment assessment of the presence of atherosclerotic plaque and diameter stenosis will be performed. Diameter stenoses will be classified as normal or near normal (< 10%), mild (10–49%), moderate (50–70%) or obstructive (> 50% stenosis in the left main stem or > 70% stenosis in other coronary arteries). Incidental findings (cardiac non-coronary and extracardiac) on CT will be documented in clinical reports and notified to responsible clinicians as per local practice.

### Management recommendations

**Fig. 1:**
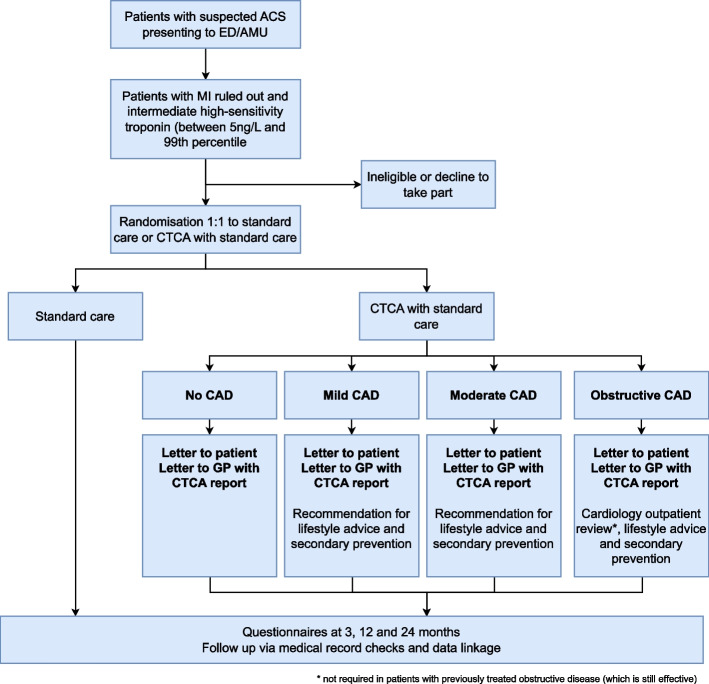
Study design overview

Following CTCA reporting, results will be reviewed by members of the research team as soon as possible and a report forwarded to the participant, primary care physician and other healthcare professionals involved in clinical care as appropriate (Fig. 1). In patients with normal or near normal (< 10% stenosis) coronary arteries, no specific action will be required. However, for patients with mild (10–49%) or moderate (50–70%) non-obstructive coronary artery disease, the report will include recommendations to commence secondary preventative medical therapy (statin therapy alone or a combination of antiplatelet and statin therapy, respectively). For patients found to have untreated obstructive coronary artery disease (> 50% stenosis in the left main stem or > 70% stenosis in other coronary arteries), the report will include recommendations to provide lifestyle advice and commence antiplatelet and statin therapy, if there are no contra-indications, and they will also be offered a cardiology outpatient consultation (either face to face or telephone based) in accordance with local NHS policy, ideally within 2 weeks of the CTCA scan result.

### Adverse events

Patients attending the scanning visit will be asked to contact the research team if they experience any untoward effects within 48 h of the CTCA scan. The research team will assess seriousness, expectedness, severity and causality of the adverse event. All serious adverse events will be reported immediately or within 24 h to the sponsors.

### Long term follow-up assessments

All patients will be followed up until at least the required number of primary outcome events have accrued. This is expected to be a minimum of 24 months for each participant and a median of 36 months. Follow-up data will be collected remotely from medical records (and any future data linkage that may be necessary). Both the short version of the Seattle Angina Questionnaire (SAQ-7) and the European Quality of Life Five Dimension (EQ-5D-5L) questionnaire will be further administered at 3 months, 12 months and 24 months after randomisation. The SAQ-7 questionnaire has been validated in patients with coronary artery disease and shown to be sensitive to therapeutic interventions [23]. The EQ-5D-5L is a validated questionnaire measuring self-reported health status over time [24]. A lifestyle questionnaire recording body mass index, physical activity, smoking, diet, blood pressure, cholesterol and blood glucose will be administered together with the SAQ-7 and EQ-5D-5L questionnaires 24 months after randomisation (Table 2).

**Table 2:**
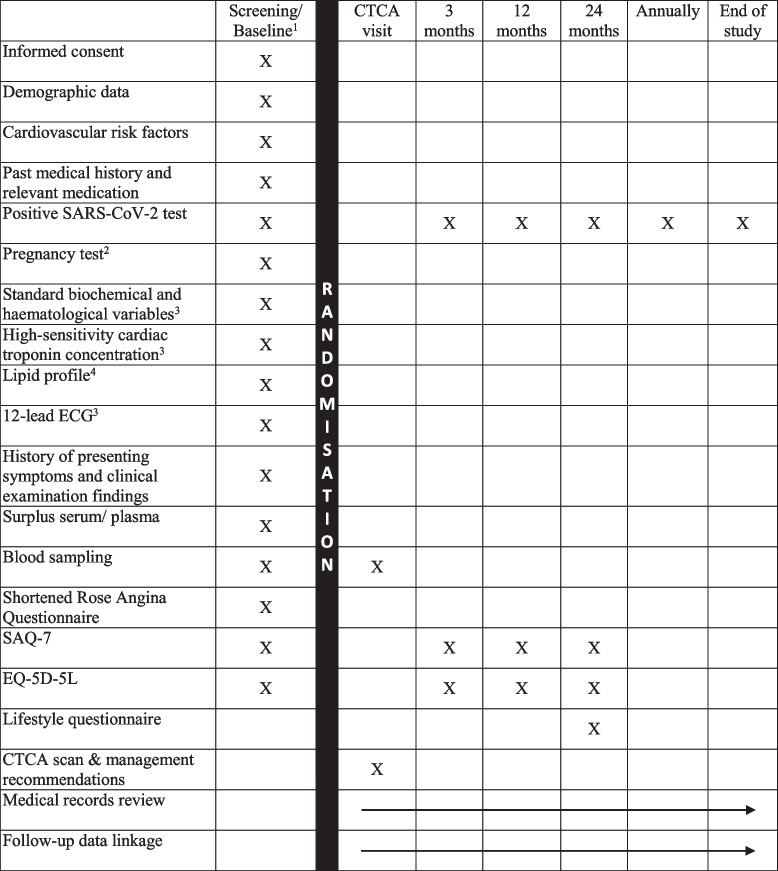
Schedule of assessments

^1^Performed either in ED/AMU or by phone

^2^To be taken if participants are uncertain of their pregnancy status

^3^As part of routine index hospital attendance

^4^Can be measured from surplus serum if not measured as part of routine hospital attendance

Data collection for outcomes will be performed by the research team at each site. All members of the research team involved in data collection will be appropriately trained and signed off by the site principal investigator on the delegation log as per Good Clinical Practice. All study data will be recorded on an electronic case report form held on a secure server. Data quality for all study outcomes will be monitored by the Edinburgh Clinical Trials Unit (ECTU) study database and data management team. Questionnaire completion will be encouraged with phone calls, letters or emails depending on the participants’ preference. Patients who subsequently decide to revoke their consent will be given the option to discontinue questionnaires or remote follow-up. This will be recorded as a change of status in the study database.

### Sample size calculation

Based on the event rate in the High-Sensitivity Troponin in the Evaluation of patients with suspected Acute Coronary Syndrome (High-STEACS) trial [19], myocardial infarction or cardiac death will occur in 7.2% of the study population at 24 months. The Scottish COmputed Tomography of the HEART (SCOT-HEART) trial [25] reported a 41% reduction in fatal and non-fatal myocardial infarction in those randomised to CTCA (hazard ratio 0.59 [95% CI: 0.41 to 0.81]), with the treatment effect of the intervention apparent at 36 months. It is anticipated that at a median follow-up of 36 months the event rate in the standard of care arm will be 9%. In the proposed study, for 90% power at an alpha of 0.05, 1081 participants will be required in each arm to detect a 40% relative risk reduction in the primary composite endpoint. To account for drop out of 5%, the aim is to recruit at least 2270 patients to the study. With this sample size, there will be 81% and 67% power to detect a 35% and 30% relative risk reduction if the intervention is less effective. Likewise, the sample size would have 99% power to detect a 50% relative risk reduction if CTCA is as effective in the study population as in those with stable chest pain [26]. With this sample size, it is estimated that 97 primary outcome events will be accrued in the standard care arm of the trial at a median follow-up of 36 months. The data monitoring committee (DMC) will review all primary outcome events intermittently and will inform the trial steering committee (TSC) when this target has been met.

On April 25, 2022, a blinded assessment of the number of events accrued in the first 1925 participants was undertaken and the DMC concluded the trial would not reach the number of primary outcome events required by the planned follow-up date of September 2024. This was a consequence of interruptions to recruitment due to COVID-19 with most participants having less than 12 months follow-up at this point. To minimise the likelihood that follow-up will need to be extended, the DMC and TSC recommended recruitment continue beyond the sample size of 2270 patients. Recruitment of up to an additional 900 participants (total 3170 participants) was completed in May 2023. This should ensure 97 primary outcome events are accrued in the standard care arm by August 2024.

### Statistical analysis

The analysis will be based on the intention-to-treat principle and will include all outcomes occurring from randomisation to a common study end date. Follow-up will continue until 97 primary outcome events have been accrued in the standard of care arm. Primary endpoint survival times will be depicted using the Kaplan–Meier survival curves and formally assessed using a Cox proportional hazards model which will adjust for study site, age, sex and previous coronary artery disease (the variables on which the randomisation was stratified). The results will be expressed as a hazard ratio with the corresponding 95% confidence intervals and *p*-value.

Secondary outcomes will be analysed using appropriate methods—logistic regression for binary outcomes and linear regression for normally distributed continuous outcomes. Continuous outcomes that are not normally distributed will be analysed using appropriate nonparametric techniques. Subgroup analysis on the primary outcome is planned for participants: (i) sex, (ii) age, (iii) with and without a previous diagnosis of coronary artery disease at randomisation, (iv) with and without preventative therapy (statin and anti-platelet agent) at randomisation, (v) above and below the sex and assay-specific median maximum high sensitivity cardiac troponin concentration at presentation. These will be assessed by examining the effect of the intervention by subgroup interaction in the Cox regression model.

The influence of any missing data on the robustness of the findings will be examined using multiple imputation models under a missing at random assumption. All statistical analyses will be pre-specified in a comprehensive statistical analysis plan, authored by the study statisticians and agreed by the independent study oversight committees.

### Cost-effectiveness analysis

EQ-5D-5L measurements will be combined with survival times to derive an estimation of within-trial quality-adjusted life years (QALYs) for all patients. Within-trial health care costs will be estimated by applying unit costs to items of resource use (hospital admissions with length of stay, emergency department attendances, cardiac tests, and treatments). Cost-effectiveness analysis will take a health service perspective to estimate the incremental cost per QALY gained using the high-sensitivity troponin guided CTCA approach compared to usual care. Costs and QALYs beyond the trial will be estimated using decision-analysis modelling, based on adaptation of an existing model [3, 27]. Health economic analyses will be pre-specified in a comprehensive Health Economic Analysis Plan.

### Trial, ethical and governance oversight

The trial has been reviewed and approved by the South-East Scotland Research Ethics Committee (19/SS/0040). The University of Edinburgh and NHS Lothian are co-sponsoring the trial and will provide monitoring oversight. The co-sponsors do not have any role in the design of the study or collection, analysis, and interpretation of data and in writing of the manuscript. All amendments to the protocol are reviewed by the co-sponsor and site Research & Development Departments before they are added to the investigator site file. All deviations are documented in a sponsor-approved deviation log held at sites. The Edinburgh Clinical Trials Unit will manage the trial and provide oversight of the data collection and statistical analysis. A trial steering committee, data monitoring committee and project management group have been established to oversee the conduct, progress and safety of the trial. The trial steering committee and data monitoring committee meet on a 6 monthly basis whilst the project management group meet once a month. The funders of the study have no role in the study design, data collection, data management, data analysis, data interpretation, writing of the report or the decision to submit the report for publication.

## Discussion

In patients presenting to the emergency department with intermediate high-sensitivity cardiac troponin concentrations in whom myocardial infarction is ruled out, the TARGET-CTCA trial will evaluate whether early outpatient CTCA reduces subsequent myocardial infarction or cardiac death. The trial will evaluate the clinical and cost-effectiveness of using high-sensitivity cardiac troponin to target CTCA in patients at increased risk despite having myocardial infarction ruled out.

The recent update of the NICE Clinical Guideline 95 evaluated the cost utility and performance of different diagnostic modalities and now recommends CTCA as the first line investigation for all patients presenting with stable chest pain suggestive of underlying coronary artery disease [28]. CTCA has high sensitivity and low cost, making it the non-invasive test of choice in the assessment of patients with symptoms of stable chest pain due to suspected coronary artery disease. In a randomised controlled trial of patients with suspected stable angina attending an out-patient clinic (SCOT-HEART, clinicaltrial.gov NCT01149590), we reported that CTCA clarifies the diagnosis of coronary artery disease and is associated with a halving of fatal and non-fatal myocardial infarction at five years (hazard ratio: 0.59 [95% CI 0.41 to 0.84]; *p* = 0.004) through better targeting of therapies [25, 26, 29]. In the CAPP trial, patients with stable chest pain who were randomised to CTCA had markedly lower rates of unplanned re-hospitalisation with chest pain at 1 year compared to those who had exercise stress electrocardiogram (5.2% versus 0.8%; *p* = 0.009) [30]. A subsequent systematic review and meta-analysis of 16 randomised controlled trials showed a reduction in downstream myocardial infarction, hospital reattendance, and follow-up testing when CTCA is performed to diagnose coronary artery disease compared to stress testing in this setting [31].

The role of CTCA in the assessment of patients with acute chest pain is less clear. Due to the large number of patients presenting to emergency departments worldwide every year, the use of further investigations to assess patients in whom the diagnosis of myocardial infarction is ruled out need to be both clinically efficacious and cost-effective. Economic modelling suggests that CTCA may be the most cost-effective modality for the diagnosis of important coronary artery disease in patients with suspected acute coronary syndrome in whom myocardial infarction is ruled out [3]. However, the likely effectiveness of this strategy at reducing myocardial infarction or cardiovascular death is highly dependent on the risk of cardiac events in the tested population. Two previous meta-analyses [32, 33] report data on ten trials of CTCA compared with functional testing in acute chest pain, mostly delivered in the North America. None of these trials were powered for subsequent myocardial infarction or death, and all were in very low risk populations with few events and a short duration of follow-up. Most participants received additional ancillary cardiac testing in the standard care arm. These analyses showed an increase in the diagnosis of coronary artery disease, invasive coronary angiography and coronary revascularisation in those receiving CTCA. A single trial of 600 patients, the CATCH (CArdiac cT in the treatment of acute CHest pain) trial, has reported longer term follow-up at a median of 18.7 months [34]. Major adverse cardiac events were observed in five patients in the CTCA group compared with fourteen patients in the standard of care group (hazard ratio 0.36 [95% CI 0.16 to 0.95]). Differences in myocardial infarction or cardiac death were not significant. Although event rates were low, this is the only trial of CTCA in acute chest pain that has shown a reduction in clinical outcomes in patients receiving CTCA.

High-sensitivity cardiac troponin testing is already performed routinely in all patients with suspected acute coronary syndrome. As such, testing has major potential to improve the risk stratification of those patients in whom acute myocardial infarction is ruled out. Patients with intermediate cardiac troponin concentrations are at increased risk of cardiac events and are therefore more likely to benefit from further evaluation using CTCA and from secondary prevention in those found to have coronary artery disease. Currently, there is marked variation in the approach to the further evaluation and follow-up of patients after myocardial infarction has been ruled out, due to a lack of evidence from clinical trials to guide practice. Local pathways are based on expert opinion extrapolated from evidence derived in other patient populations. Consequently, current guidelines do not recommend any particular investigative approach for this large group of patients. As such, there is clinical equipoise to justify randomising patients to standard care or standard care with targeted CTCA in those with intermediate cardiac troponin concentrations.

The TARGET-CTCA trial will determine whether high-sensitivity cardiac troponin-guided CTCA can improve outcomes and reduce subsequent major adverse cardiac events in patients presenting to the emergency department who do not have myocardial infarction.

## Trial status

Recruitment began in September 2019 and has been completed on 11 May 2023. The trial opened using protocol v2.0, and the current protocol in use is v5.0.

Key amendments for the TARGET CTCA trial include:

- Amendment (September 7, 2020): Addition of new sites.
- Protocol Amendment v3.0 (March 25, 2021): Changes to data collection methods at sites, clarifications to Sect. 5.1 (identifying participants), Sect. 5.4 (withdrawals), Sect. 6.1.2 (scanning visit), Sect. 6.1.3 (management recommendations), Sect. 7 (assessments), and Sect. 8.2 (proposed analyses, introduction of COVID-19 test data).
- Amendment (August 4, 2021): Recruitment extension from September 1, 2021, to August 31, 2022, due to impact of COVID-19.
- Protocol Amendment v4.0 (July 7, 2022): Recruitment extension for 8 months until April 2023 and an increase in the recruitment target from 2270 to 3170.
- Protocol Amendment v5.0 (September 26, 2022): Introduction of a new lifestyle questionnaire at 24 months.

## Supplementary Information

**Additional file 1: Supplementary Table 1.** List of participating hospitals and sites.

**Additional file 2.**

## Data Availability

All participant data will be stored securely and identified by an anonymised study ID to maintain participants’ confidentiality. Following publication of the primary paper, a deidentified individual participant data set will be made available for data sharing purposes, subject to necessary governance approvals.

## Abbreviations

CTCA: Computed tomography coronary angiography
DMC: Data monitoring committee
ED: Emergency department
EQ-5D: European Quality of Life Five Dimension
ESC: European Society of Cardiology
GP: General practitioner
High-STEACS: High-sensitivity troponin in the evaluation of patients with suspected acute coronary syndrome trial
NICE: National Institute for Health and Care Excellence
SAQ-7: Seattle Angina Questionnaire
SCOT-HEART: The Scottish computed tomography of the heart trial
TSC: Trial steering committee
