# Supplementary material for: Troponin in acute chest pain to risk stratify and guide effective use of computed tomography coronary angiography (TARGET-CTCA): a randomised controlled trial
Source: Trials. 2023 Jun 13;24:402. doi: 10.1186/s13063-023-07431-9 (PMC10264092; doi:10.1186/s13063-023-07431-9)
Supplement: Supplementary file 1 — Additional file 1: Supplementary Table 1. List of participating hospitals and sites. [file 13063_2023_7431_MOESM1_ESM.docx]

##### supplement

**Troponin in Acute chest pain to Risk stratify and Guide EffecTive use of Computed Tomography**

**Coronary Angiography (TARGET-CTCA):**

**a randomised controlled trial**

Kuan Ken Lee, MD,^1^ David Lowe, MD,^2^ Rachel O’Brien, BN,^3^ Ryan Wereski, MD,^1^

Anda Bularga, MD,^1^ Caelan Taggart, MD,^1^ Matthew TH Lowry, MD,^1^ Amy V Ferry, PhD, ^1^

Michelle C Williams, MD,^1^ Giles Roditi, MD,^4^ John Byrne, MD,^5^ Chris Tuck BSc,^1^

Denise Cranley, BSc,^6^ Praveen Thokala, PhD,^7^ Steve Goodacre, PhD,^7^

Catriona Keerie, MSc,^6^ John Norrie, MSc,^6^ David E Newby, MD,^1^

Alasdair J Gray, MD,^3,8^ Nicholas L Mills, MD.^1,8^

^1^ BHF Centre for Cardiovascular Science, University of Edinburgh, Edinburgh, UK.

^2^ Department of Emergency Medicine, Queen Elizabeth University Hospital, NHS Greater Glasgow and Clyde, Glasgow, UK.

^3^ Department of Emergency Medicine, Emergency Medicine Research Group, Royal Infirmary of Edinburgh, Edinburgh, UK.

^4^ Institute of Cardiovascular and Medical Sciences, Glasgow University, Glasgow, UK.

^5^ Department of Cardiology, Queen Elizabeth University Hospital, NHS Greater Glasgow and Clyde, Glasgow, UK.

^6^ Edinburgh Clinical Trials Unit, Usher Institute, University of Edinburgh, Edinburgh, UK.

^7^ School of Health and Related Research (ScHARR), University of Sheffield, Sheffield, UK.

^8^ Usher Institute of Population Health Sciences and Informatics, University of Edinburgh, Edinburgh, UK.

**Corresponding Author:**

Professor Nicholas L Mills

BHF/University Centre for Cardiovascular Science

The University of Edinburgh

Edinburgh EH16 4SA

United Kingdom

Telephone: 0044 131 242 6515

E-mail: [nick.mills@ed.ac.uk](mailto:nick.mills@ed.ac.uk)

**Supplementary Table:** 1

**Supplementary Table 1.** List of participating hospitals and sites

| **Study site** | **Hospital** |
| --- | --- |
| NHS Lothian | Royal Infirmary of Edinburgh |
| NHS Lothian | St John’s Hospital |
| NHS Lothian | Western General Hospital |
| NHS Lothian | Victoria Hospital, Kirkcaldy (Participant Identification Centre, PIC) |
| NHS Greater Glasgow and Clyde | Queen Elizabeth University Hospital |
| NHS Greater Glasgow and Clyde | Royal Alexandra Hospital |
| NHS Greater Glasgow and Clyde | Glasgow Royal Infirmary |
| Lewisham & Greenwich NHS | University Hospital Lewisham |
| Royal Berkshire NHS | Royal Berkshire Hospital |
| Milton Keynes NHS | Milton Keynes University Hospital |
| Torbay & South Devon NHS | Torbay Hospital |
| North Tees NHS | University Hospital of North Tees |
| Plymouth NHS Trust | Derriford Hospital |
| Southampton NHS Foundation trust | Southampton General Hospital |
